# Rapid Determination of Saponins in the Honey-Fried Processing of Rhizoma Cimicifugae by Near Infrared Diffuse Reflectance Spectroscopy

**DOI:** 10.3390/molecules23071617

**Published:** 2018-07-03

**Authors:** Lun Wu, Yang Su, Haoran Yu, Xiuhui Qian, Xueting Zhang, Qiuhong Wang, Haixue Kuang, Genhong Cheng

**Affiliations:** 1 Institute of Traditional Chinese Medicine, Heilongjiang University of Chinese Medicine, Harbin 150040, China; wulun2012@163.com (L.W.); yuhaoran94@163.com (H.Y.); qxh_1027@126.com (X.Q.); 18845055847@163.com (X.Z.); 2 School of Pharmacy, Heilongjiang University of Chinese Medicine, Key Laboratory of Medicinal Materials, Chinese Academy of Sciences, Harbin 150040, China; suyanggo@163.com; 3 School of Traditional Chinese Medicine, Guangdong Pharmaceutical University, Guangzhou 510000, China; 4 Faculty of Microbiology and Immunogenetics, University of California, Los Angeles, CA 90095, USA; gcheng@mednet.ucla.edu

**Keywords:** Rhizoma Cimicifugae, honey-fried processing, near-infrared spectroscopy, quantitative model

## Abstract

Objective: A model of Near Infrared Diffuse Reflectance Spectroscopy (NIR-DRS) was established for the first time to determine the content of Shengmaxinside I in the honey-fried processing of Rhizoma Cimicifugae. Methods: Shengmaxinside I content was determined by high-performance liquid chromatography (HPLC), and the data of the honey-fried processing of Rhizoma Cimicifugae samples from different batches of different origins by NIR-DRS were collected by TQ Analyst 8.0. Partial Least Squares (PLS) analysis was used to establish a near-infrared quantitative model. Results: The determination coefficient R^2^ was 0.9878. The Cross-Validation Root Mean Square Error (RMSECV) was 0.0193%, validating the model with a validation set. The Root Mean Square Error of Prediction (RMSEP) was 0.1064%. The ratio of the standard deviation for the validation samples to the standard error of prediction (RPD) was 5.5130. Conclusion: This method is convenient and efficient, and the experimentally established model has good prediction ability, and can be used for the rapid determination of Shengmaxinside I content in the honey-fried processing of Rhizoma Cimicifugae.

## 1. Introduction

Rhizoma Cimicifugae is primarily derived from *Cimicifuga heracleifolia Kom*., *Cimicifuga dahurica (Turcz.) Maxim.*, or *Cimicifuga foetida L*. [1]. It is a kind of cool and deconstructive drug commonly used in Chinese traditional medicine. It was first recorded in “Sheng Nong’s herbal classic” and appeared as a “top grade product” drug. It has the effect of clearing rash, heat, and detoxifying and lifting yang [1]. Cimicifugae is suitable for growing in mountains, forests, and roadside grasslands about 2000 m above sea level, and their main producing areas are in the three northeastern provinces of China [2]. *Cimicifuga Simplex* Wormsk is a perennial herb suitable for growing in warm, humid climates and slightly acidic humus. In the cultivation of Cimicifugae, it is necessary to prevent soil from drought. In addition, the seedlings of Cimicifugae are afraid of strong light, but its flowering period requires sufficient sunlight [3].

Up to now, it is reported that Cimicifugae mainly contains triterpenoids, triterpene saponins, phenylpropanoids [4], chromone, volatile oil, and other compounds [5]. One of the triterpene saponins compound is Shengmaxinside Im which has antipyretic and analgesic effects [4]. In addition, according to recent studies, Cimicifugae saponins also have anti-tumor, anti-inflammatory, anti-viral, hepatoprotective, anti-nucleoside transport, anti-osteoporosis, and antioxidant effects [6].

According to the needs of clinical medical treatment, traditional Chinese medicines must be processed before being used, so as to improve the curative effect and expand the scope of application. In addition, Chinese medicine practitioners believe that honey is sweet and tasteful, and it nourishes the lungs and eases bowel movements. After processing with honey, Chinese medicines can have enhanced effects of replenishing spleen Qi, moistening the lungs, and relieving cough, and it can improve the flavoring of the herbs [7]. In clinical applications, Rhizoma Cimicifuga is often processed with honey to enhance its properties, making it more suitable for the treatment of rectal prolapse, uterine prolapse, and other conditions.

However, so far, there is still not a perfect method to evaluate the active compounds of Rhizoma Cimicifuga that has been fried with honey. The traditional method of determining Shengmaxinside I content is high performance liquid chromatography (HPLC) or ultra-high performance liquid chromatography with quadrupole-time-of-flight mass spectrometry [8]. However, the previous extraction process is cumbersome and it takes a long time [9], which does not meet the requirements for rapid analysis of large quantities of medicinal materials. 

In recent years, Near Infrared Diffuse Reflectance Spectroscopy has had broad application in many fields. It has the advantages of being simple and convenient to use, provides fast analysis, and causes no damage to samples, which is an environmentally-friendly, fast, and convenient non-invasive analysis technology [10]. For example, it is used to rapidly measure the moisture content of coffee beans [11], to measure the content of heavy metals in soil [12], and to determine copper and zinc contaminants in Ludwigia prostrata Roxb, etc. [13]. What’s more, it has also achieved rapid development in the analysis of traditional Chinese medicine, for example, six active ingredients of Cistanche deserticola can be measured at the same time by this technique [14]. The technique comprehensively reflects the overall information of medicinal herbs and facilitates macro-cluster analysis [15]. It has been reported that saponins can be detected by near-infrared diffuse reflectance spectroscopy, including Ginsenosides [16], Notoginsenoside [17], etc. It is feasible to use NIR spectroscopy to analyze the material in Cimicifuga. As it reported, NIR-DRS can determine polyphenols and triterpene glycosides [18]. 

In previous studies, we successfully established a model for the determination of isoferulic acid in the Rhizoma Cimicifugae using near-infrared spectroscopy [19]. The purpose of this study was to quickly use the near-infrared spectroscopy technique to measure the content of isoferrite in Cimicifugae. The detection of saponin content by near-infrared spectroscopy has great practical significance.

## 2. Materials and Methods

### 2.1. Materials

An Antaris II Fourier Transform Near Infrared Spectrometer (Thermo Scientific, Inc., Waltham, MA, USA); Agilent 1200 HPLC (Agilent Technologies, Inc., Palo Alto, CA, USA); 1260 Infinity Evaporation Detector (Agilent Technologies, Inc., Palo Alto, CA, USA) G2070BA Workstation; Rotary Evaporator N-1100 (EYELA Shanghai Ai Lang Instrument Co., Shanghai, Ltd., Shanghai, China) were used. HPLC grade acetonitrile and formic acid was purchased from Fisher Chemicals (Fisher Scientific, Waltham, MA, USA). All other reagents were of analytical grade.

Cimicifugaes were purchased from different batches of different production areas around China. A total of 150 batches were produced in Anhui Bozhou, Datong Shanxi, Gansu, Yunnan, Shanxi, Sichuan, Henan, Northeast, and the other places shown in Table 1.

**Figure 1:**
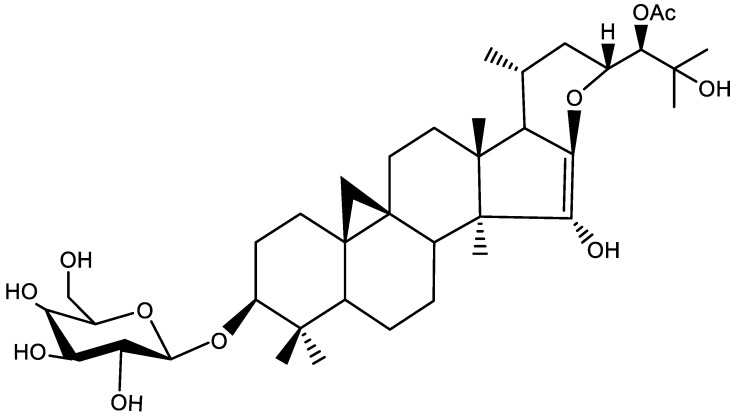
Structure of Shengmaxinside I.

The standard product Shengmaxinside I (16,17-didehydro-24*S*-*O*-acetyl hydroshengmanol-3-*O*-*β*-d-galactopyranoside) was prepared in the laboratory and its purity reached 95%. The structure is shown in Figure 1. The ^1^H-NMR shows that cyclopropane matrix signals δ_H_ 0.16 (1H, d, *J* = 3.8 Hz) and δ_H_ 0.59 (1H, d, *J* = 3.8 Hz), and a methylene proton signal can be seen in the high field region. *J* = 6.8 Hz, 6 methyl proton signals, 4 oxo-methyl proton signals, 1 acetyl-matrix signal, and 1 sugar-terminal proton signal, suggesting that the backbone is 9, 19-Cyclopentane triterpenoid saponin. The ^13^C-NMR shows that from the carbon signals of δ_C_ 121.4 and 151.0, the double bond is located at C-16 and 17 positions, that is, the D ring of 25 is dehydrated at C-16 and C-17 positions to form a double bond, which is a hydroshengmanol-type ring of pineapple beesin triterpene saponins.

### 2.2. Determination of Shengmaxinside I by HPLC

#### 2.2.1. Chromatographic Conditions 

C18 column kromasil (200 mm × 4.6 mm, 5 μm), column temperature 25 °C. Mobile phase: gradient elution of acetonitrile (A) and 0.1% aqueous formic acid (B) (0 to 5 min, 95% B; 5 to 20 min, 95% to 60% B; 20 to 36 min, 60 to 45% B; 36~41 min, 45% B; 41~45 min, 45~0% B), flow rate: 1.0 mL·min^−1^, detection by evaporative light scattering detector, drift tube temperature 80 °C, carrier pressure 3.50 bar, and the number of theoretical plates is no less than 5000.

#### 2.2.2. Determination Methods 

A 20 μL solution of Shengmaxinside I was injected into the HPLC for 45 min. The test solution was treated in the same way.

#### 2.2.3. Preparation of the Reference Solution 

A 1.53 mg sample of Shengmaxinside I was dissolved in 10 mL 70% ethanol solution to obtain the standard solution.

#### 2.2.4. Preparation of the Test Solution 

We precisely weighed Rhizoma Cimicifugae 1.000 g in a round bottom flask. After, we added 40 mL 70% ethanol solution and heated, refluxed, and extracted it for 2 h, and then it was filtered. Then, we added 70% ethanol solution 40 mL again and repeated the above operation two times. The solutions were merged and then evaporated. The resulting precipitate was dissolved in 50 mL 70% ethanol and was filtered with a 0.22 μm microporous membrane to get the test solution.

#### 2.2.5. Investigation of Linear Relationship 

We accurately absorbed 4.0, 6.0, 8.0, 10.0, 12.0 and 14.0 μL of Shengmaxinside I standard solution into a 20 μL of sample was injected, and the peak area (Y) was plotted against the concentration of the reference solution (X). The standard curve of Shengmaxinside I was obtained as y = 1.5609x + 5.1509 (r = 0.999), showing a good linear relationship between concentration and peak area in the range of 0.0306 mg to 0.1071 mg.

#### 2.2.6. Precision Experiment 

Twenty μL of the reference solution was precisely pipetted and continuously injected 5 times according to the chromatographic conditions above. The RSD value of Shengmaxinside I was 0.91%, calculated from the peak area, indicating that the precision of the instrument was good. 

#### 2.2.7. Stability Experiment 

For the same test solution, the peak areas were measured at 0, 4, 8, 12, and 24 h, respectively, according to the chromatographic conditions above, and the RSD was 1.79%, indicating that the test solution was stable within 24 h.

#### 2.2.8. Repetitive Experiment 

5 samples from the same batch were accurately weighed and prepared according to the method of the test solution. According to the chromatographic conditions above, the average content was determined to be 0.425%, and the RSD was 0.89%, indicating that the method had good repeatability.

#### 2.2.9. Sample Recovery Experiment 

We precisely absorbed the sample solution and added the standard solution of high-, middle-, and low-concentration gradients of Shengmaxinside I, and then injected 20 μL of each sample to determine the recovery of the corresponding components according to the above chromatographic conditions. The average recovery rate was 99.14% and the RSD was 1.75%.

### 2.3. Spectral Acquisition

**Figure 2:**
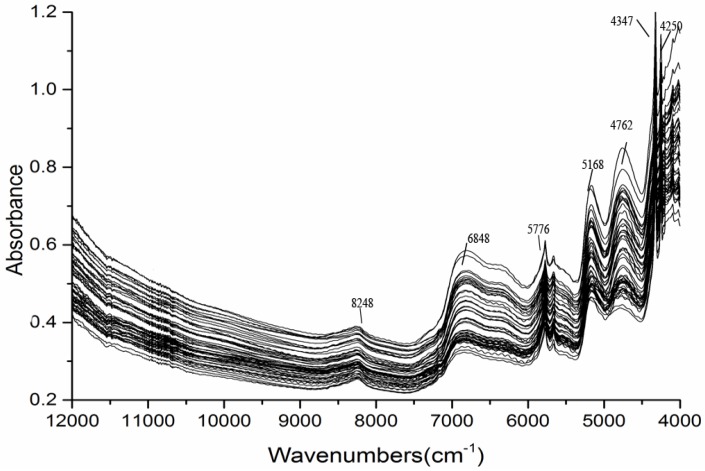
NIRS spectra of 150 batches of Rhizoma Cimicifugae.

The scanning spectrum range was 12,000–4000 cm^−1^ with a resolution ratio of 0.5 cm^−1^. Two grams of the medicinal powder was placed in a quartz sample cup. Each batch of the 150 batches was reloaded and scanned 3 times. The average was then taken. The detected spectra were superimposed (150 in all), as shown in Figure 2.

### 2.4. Near-Infrared Model Evaluation Index and Establishment of Modeling Methods

When establishing a quantitative analysis model of NIR-DRS analysis, TQ Analyst 8.0 data processing software is used. Multi-linear regression (MLR), Step-wise Multi-linear regression (SMLR), Principal component regression Principal Component Regression (PCR), Partial Least Squares (PLS), and other methods can help establish the model. In the past, MLR was used for correction data. However, the PCR and PLS have been widely used because the new generation of NIR spectrometers can collect spectra in all NIR bands. For the determination of complex sample systems, both PCR and PLS can be applied. Compared with MLR, PCR is slower and the understanding of the model is less intuitive. The most important thing is that it can identify the main factors that affect the system and can solve the problems of colinearity and the number of variables in the linear regression analysis. Relative to the former, PLS establishes a more robust model with the widest range of applications, and the resulting eigenvectors are directly related to sample properties [20]. Therefore, PLS was determined as an analytical method after comprehensive comparison.

While establishing a quantitative analysis model, the performance of the model has to be evaluated. In the NIR-DRS analysis, there are two inspection indicators that are commonly used for quantitative analysis of model results. One is the Cross-Validation Root Mean Square Error (RMSECV), and the other is the determination coefficient (R^2^). The closer the R^2^ value is to 1, the better the correlation between the predicted value of the model and the measured value of the sample; and the smaller the RMSECV, the more stable the model performance and the higher the accuracy [21].

Verification samples are used to verify the NIR-DRS quantitative model by taking the Root Mean Square Error of Prediction (RMSEP) as the inspection index. The RPD is the ratio of the standard deviation for the validation samples to the standard error of prediction, which is also the inspection index.

### 2.5. Correction Set and Verification Set Division

One hundred and fifty spectra were obtained by the Antaris II Fourier Transform Near Infrared Spectrometer. Besides, the TQ Analyst 8.0 data processing software randomly selected 120 representative spectra as calibration sets and 30 as validation sets. The correction set content range is 0.12%–0.52% (*w*/*w*), and the validation set content range is 0.14%–0.50% (*w*/*w*). Because the content of Shengmaxinside I in the verification sample set is within the calibration set content range, this calibration set and verification set can be used for modeling.

## 3. Results and Conclusions

### 3.1. Content of Shengmaxinside I in Rhizoma Cimicifugae Extract

**Figure 3:**
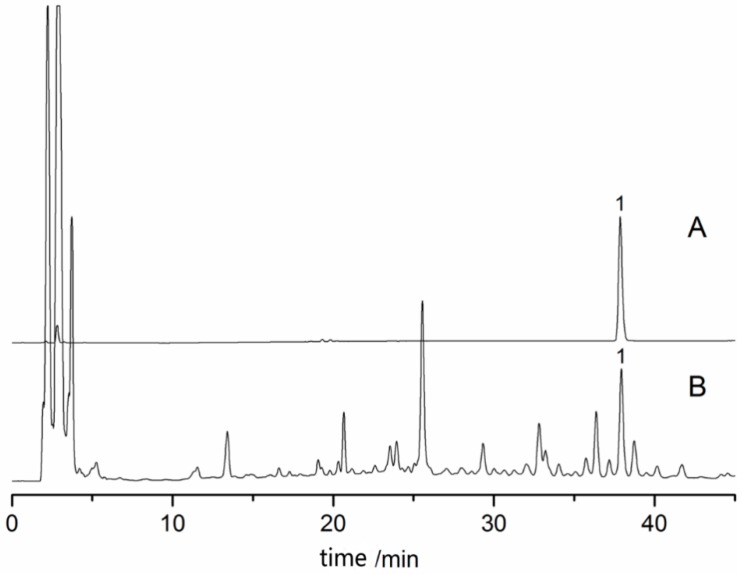
HPLC chromatogram of Shengmaxinside I content from Rhizoma Cimicifugae.

According to the above method, the content of Shengmaxinside I in the sample of Rhizoma Cimicifugae was determined. The chromatogram is shown in Figure 3. As it shown in the Figure 3: A. Reference substance; B. Sample; 1. Shengmaxinside I.

In order to calculate the content of Shengmaxinside I in Rhizoma Cimicifugae, the sample content is calculated based on dry products. Each batch of samples is measured in parallel and averaged. The results showed that the 150 samples ranged from 0.12% to 0.52% (*w*/*w*), shown in Table 2.

### 3.2. Establishment and Selection of Near-Infrared Quantitative Model

#### 3.2.1. Investigation of Near-Infrared Method 

We weighed 2 g powder of Cimicifugae and collected the signal 9 times under the same spectral conditions to calculate the precision. The RSD was 1.09%.

From Figure 2, we can get the following information. There are a few characteristic peaks absorbed in the wavenumber band of 8500–12,000 cm^−1^. In the 4000–4200 cm^−1^ band, fiber absorption is not suitable for modeling because it contains more noise. There are distinct characteristic absorption peaks within 4500–8500 cm^−1^, and therefore, selective modeling is selected within this band range.

According to the raw NIR spectra (Figure 2) of 150 samples at wavenumbers ranging from 4000 to 10,000 cm^−1^, several characteristic absorption peaks can be seen. For example, 4250 cm^−1^ is the C–H stretch/C–H deformation, 4357 cm^−1^ is the stretch and bending combination of –CH_2_, 4762 cm^−1^ is the performance of stretching vibration of C–C and C=C bonds, 5168 cm^−1^ due to the second overtone of C=O stretching bands of acetyl and maybe also the stretching and deformation of O–H bonds, 5776 cm^−1^ results from first overtone of stretching C–H bonds, and 6848 cm^−1^ is the O–H stretching first overtone. In addition, the second overtone of C–H stretching arises around 8248 cm^−1^ [22,23,24,25]. These signals could reflect the chemical information of Shengmaxinside I.

#### 3.2.2. Selection of Spectral Pretreatment Methods 

In the process of sample collection, due to the influence of the sample’s grain size, color, and instrument response, the near-infrared raw spectra often contain factors that are not related to the nature of the sample to be measured, resulting in interference such as near-infrared spectral shift or drift. Thus, it is necessary to carry out pretreatment [2]. In terms of RMSECV, RMSEP, and the value of R^2^ as indicators, we need to examine the spectra, first derivative (FD), second derivative (SD), multiple scatter correction (MSC), and normal variable correction (SNV), classic Savitzky–Golay (SG) filtering, Norris derivative filtering, and more other preprocessing methods. With the data processing as the index, the R^2^ and the RMSECV are comprehensively examined. As shown in Table 3, the pretreatment method for determining the Shengmaxinside I model was MSC + SD + SG.

#### 3.2.3. Spectral Range Selection 

Based on the absorption spectra of each hydrogen-containing group, the content of the materials in the samples were obtained. However, the information contained in the different spectral ranges is different. Therefore, a more accurate quantitative model can be obtained by selecting an appropriate spectral interval model [26]. In this research, the spectrum of Shengmaxinside I standard product was compared with multiple ranges, and R^2^, RMSECV, RPD, and RMSEP were selected as comprehensive indicators to examine. As shown in Table 4, the spectrum used for modeling was finally determined and the intervals are 5200–6700 cm^−1^ and 7700–8800 cm^−1^. There are few characteristic peaks absorbed in the wavelength band of 8500–12,000 cm^−1^.

#### 3.2.4. The Choice of the Best Main Factor 

When establishing a near-infrared model, it is particularly important to determine the number of best principal factors involved in modeling. If the main factors are too few, much useful information of the original spectrum will be lost, and the fitting will be insufficient, which will reduce the prediction accuracy of the model; if too many, the measurement noise will be excessively high. The phenomenon of overfitting appears to reduce the predictive ability of the model [23]. The number of PLS factors can be determined from Prediction Residual Sum of Squares (PRESS) and RMSECV. The number of PLS factors is determined to be 6.

#### 3.2.5. Verification of the Model 

The model is established by the PLS method through TQ Analyst 8.0 software, and spectral preprocess by MSC + SD + SG. The spectral range is 5200–6700 cm^−1^ and 7700–8800 cm^−1^, and the number of factors was 6. The determination coefficient of the model of Shengmaxinside I was 0.9878%, the corrected mean square error (RMSECV) was 0.0193%, and RPD was 5.5130, as shown in Table 5.

**Figure 4:**
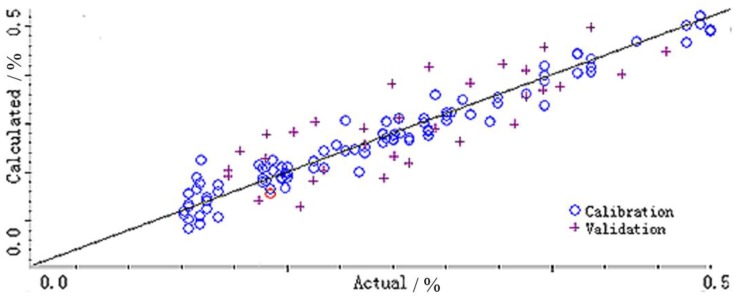
Correlation between predicted content and authentic content. 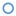 represents calibration; + represents validation.

The experimental samples were selected from 500 samples and selected by the WinISI 4.3 software to obtain 150 samples which obey the “boxcar” distribution, and the content of each sample is shown in Table 2. The correlation between the predicted content and the authentic content is shown in Figure 4.

**Figure 5:**
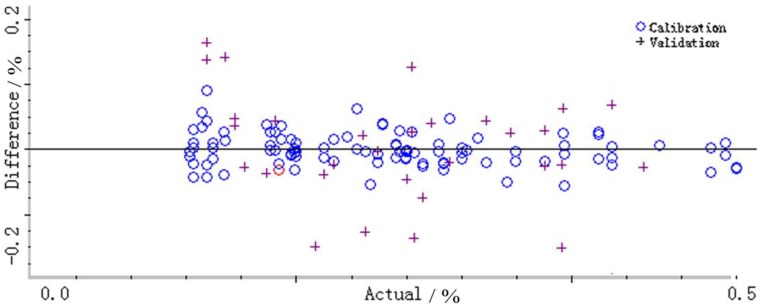
Error distribution. 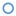 represents calibration; + represents validation.

One hundred and fifty NIR-DRS datasets belonging to the verification set were substituted into the model, and the error distribution map and relative trend comparison chart between the validation set NIR-DRS prediction value and the actual measured value of the reference method were obtained. The model has a predicted mean square error RMSEP of 0.1064%, which is shown in Figure 5, and the model has good predictive power.

## 4. Discussion

During the early stage of this experiment, we explored the method of determining Shengmaxinside I in Cimicifugae by HPLC, which has good precision and accuracy. Based on this, we extracted the honeydew medicinal materials from different batches of different origins and determined the content of Shengmaxinside I. The results showed that the differences in the regions affected the content of these components, which provided the basis for the extensive applicability of subsequent quantitative models. This is the first time a model to determine the content of traditional Chinese medicine in honey processing by NIR-DRS has been built, so this conclusion provides an exemplary role for other research on Chinese medicine processing.

Using near-infrared spectroscopy combined with chemometric methods, a quantitative model of Dhengmaxinside I in Cimicifugae was established. In the process of model building, most rely on analysis software and statistical methods to reduce the error caused by human operation. To a certain extent, it predicts the reliability and accuracy of the results, and improves the efficiency of sample measurement. The experimental results show that the established model has good predictive ability. However, in the actual production and analysis process, a faster method is needed because as long as the NIR spectra are obtained by scanning powder samples in the established near-infrared quantitative analysis model, the content of Dhengmaxinside I in the Cimicifugae samples can be quickly predicted. Thus, in this research, a near-infrared quantitative model of Shengmaxinside I was established by NIR-DRS combined with chemometric methods. Comparing the two methods of content determination, NIR-DRS is more convenient and faster than HPLC. It is suitable for determination of large batches of medicinal materials without damaging the sample and is safe and environmentally friendly. However, a limitation is that it requires the establishment of a quantitative model, and a determined chemical measurement method is required as a bedding, which is not suitable for the determination of a small sample or a small dose of an unmodeled drug. Furthermore, the Cimicifuga sources used in the establishment of this model are all from China, so there may be limitations in the analysis of Cimicifuga from other regions.

Although near-infrared spectroscopy technology is convenient and quick, the process of establishing the model in the early stage is complex and needs to be based on traditional chemical methods and cannot be completely replaced. Therefore, in order to fully exploit the strengths of the NIR-DRS method, follow-up research will be devoted to the establishment of an extensive library of traditional Chinese medicine near infrared models.

## Figures and Tables

**Table 1:** Origin of Rhizoma Cimicifugae.

Number	Origin
**1–30**	Northeast of China
**30–50**	Anhui Bozhou, China
**50–75**	Datong Shanxi, China
**75–103**	Gansu, China
**103–120**	Yunnan, China
**120–130**	Shanxi, China
**130–145**	Sichuan, China
**145–150**	Sichuan, China

**Table 2:** Cimicifuga Content Determination Results (n = 150).

Number	Peak Area	Percentage	Number	Peak Area	Percentage
**Sample 1**	848.8	0.1885%	**Sample 76**	1633.7	0.2868%
**Sample 2**	465.7	0.1283%	**Sample 77**	1563.2	0.2788%
**Sample 3**	665.4	0.1613%	**Sample 78**	1111.5	0.2241%
**Sample 4**	679.1	0.1634%	**Sample 79**	753.2	0.1746%
**Sample 5**	654.1	0.1595%	**Sample 80**	1028.3	0.2132%
**Sample 6**	482.8	0.1313%	**Sample 81**	1556.3	0.2780%
**Sample 7**	445.2	0.1247%	**Sample 82**	1432.5	0.2636%
**Sample 8**	666.4	0.1615%	**Sample 83**	1775.3	0.3025%
**Sample 9**	629.1	0.1556%	**Sample 84**	981.2	0.2069%
**Sample 10**	852.9	0.1891%	**Sample 85**	1092.5	0.2216%
**Sample 11**	698.9	0.1665%	**Sample 86**	1137.6	0.2274%
**Sample 12**	682.4	0.1639%	**Sample 87**	1523.4	0.2742%
**Sample 13**	745.4	0.1735%	**Sample 88**	1966.2	0.3229%
**Sample 14**	878.7	0.1928%	**Sample 89**	1785.2	0.3035%
**Sample 15**	830.7	0.1859%	**Sample 90**	2114.9	0.3384%
**Sample 16**	693.4	0.1656%	**Sample 91**	1137.9	0.2275%
**Sample 17**	679.1	0.1634%	**Sample 92**	1571.1	0.2797%
**Sample 18**	845.3	0.1880%	**Sample 93**	2538.7	0.3804%
**Sample 19**	783.5	0.1791%	**Sample 94**	2323.2	0.3594%
**Sample 20**	699.7	0.1666%	**Sample 95**	2302.5	0.3573%
**Sample 21**	838.8	0.1871%	**Sample 96**	1233.5	0.2395%
**Sample 22**	779.7	0.1785%	**Sample 97**	1556.2	0.2780%
**Sample 23**	682.4	0.1639%	**Sample 98**	19,586.3	1.4083%
**Sample 24**	629.1	0.1556%	**Sample 99**	1445.8	0.2652%
**Sample 25**	677.9	0.1632%	**Sample 100**	1863.5	0.3120%
**Sample 26**	894.3	0.1949%	**Sample 101**	1765.3	0.3014%
**Sample 27**	823.7	0.1849%	**Sample 102**	1554.2	0.2778%
**Sample 28**	663.4	0.1610%	**Sample 103**	2635.8	0.3896%
**Sample 29**	755.3	0.1749%	**Sample 104**	2344.1	0.3614%
**Sample 30**	822.9	0.1848%	**Sample 105**	2357.6	0.3628%
**Sample 31**	896.3	0.1952%	**Sample 106**	2212.4	0.3483%
**Sample 32**	921.5	0.1987%	**Sample 107**	1763.2	0.3011%
**Sample 33**	781.3	0.1788%	**Sample 108**	1723.4	0.2968%
**Sample 34**	2000.3	0.3265%	**Sample 109**	2563.2	0.3827%
**Sample 35**	1785.2	0.3035%	**Sample 110**	2156.3	0.3426%
**Sample 36**	931.2	0.2001%	**Sample 111**	1239.5	0.2403%
**Sample 37**	852.3	0.1890%	**Sample 112**	1452.8	0.2660%
**Sample 38**	763.2	0.1761%	**Sample 113**	1453.2	0.2661%
**Sample 39**	618.5	0.1539%	**Sample 114**	1569.5	0.2795%
**Sample 40**	1295.4	0.2472%	**Sample 115**	2356.4	0.3626%
**Sample 41**	1956.2	0.3219%	**Sample 116**	1456.7	0.2665%
**Sample 42**	1532.3	0.2752%	**Sample 117**	3566.5	0.4729%
**Sample 43**	765.4	0.1764%	**Sample 118**	1752.5	0.3000%
**Sample 44**	2150.5	0.3420%	**Sample 119**	1456.2	0.2664%
**Sample 45**	1005.6	0.2102%	**Sample 120**	2681.1	0.3939%
**Sample 46**	935.2	0.2006%	**Sample 121**	2959.6	0.4196%
**Sample 47**	1491.1	0.2705%	**Sample 122**	3070.9	0.4297%
**Sample 48**	1863.5	0.3120%	**Sample 123**	4137.4	0.5201%
**Sample 49**	1632.2	0.2866%	**Sample 124**	4065.1	0.5143%
**Sample 50**	653.2	0.1594%	**Sample 125**	3907.1	0.5014%
**Sample 51**	1238.5	0.2402%	**Sample 126**	1032	0.2137%
**Sample 52**	1653.2	0.2890%	**Sample 127**	4034.4	0.5118%
**Sample 53**	934.2	0.2005%	**Sample 128**	2258.1	0.3529%
**Sample 54**	1456.3	0.2664%	**Sample 129**	1352.7	0.2541%
**Sample 55**	1943.8	0.3206%	**Sample 130**	1978.3	0.3242%
**Sample 56**	1523.2	0.2742%	**Sample 131**	2054.1	0.3321%
**Sample 57**	1522.3	0.2741%	**Sample 132**	1784.2	0.3034%
**Sample 58**	852.3	0.1890%	**Sample 133**	2689.7	0.3947%
**Sample 59**	1456.2	0.2664%	**Sample 134**	1212.5	0.2369%
**Sample 60**	1681.7	0.2922%	**Sample 135**	1859	0.3115%
**Sample 61**	931.1	0.2000%	**Sample 136**	1611.3	0.2843%
**Sample 62**	1079.9	0.2200%	**Sample 137**	849.1	0.1886%
**Sample 63**	1423.3	0.2625%	**Sample 138**	2675.1	0.3933%
**Sample 64**	1266.7	0.2436%	**Sample 139**	3460.7	0.4639%
**Sample 65**	1815.1	0.3068%	**Sample 140**	1623	0.2856%
**Sample 66**	1756.3	0.3004%	**Sample 141**	1322.8	0.2505%
**Sample 67**	1023.6	0.2126%	**Sample 142**	1578	0.2805%
**Sample 68**	953.2	0.2031%	**Sample 143**	1530	0.2750%
**Sample 69**	1563.2	0.2788%	**Sample 144**	670.1	0.1620%
**Sample 70**	1456.3	0.2664%	**Sample 145**	727.7	0.1708%
**Sample 71**	1955.2	0.3218%	**Sample 146**	702	0.1669%
**Sample 72**	1756.4	0.3004%	**Sample 147**	900.9	0.1959%
**Sample 73**	886.5	0.1938%	**Sample 148**	873.5	0.1920%
**Sample 74**	1069.2	0.2186%	**Sample 149**	1025.6	0.2128%
**Sample 75**	1522.4	0.2741%	**Sample 150**	735.2	0.1719%

**Table 3:** The influence of different pretreatment methods on the quantitative model (4500–8500 cm^−1^).

Method	R^2^	RMSECV	Method	R^2^	RMSECV
**MSC + Spectrum**	0.75194	0.0489	**SNV + Spectrum**	0.75953	0.0470
**MSC + FD**	0.76722	0.0447	**SNV + FD**	0.77186	0.0442
**MSC + SD**	0.80167	0.0405	**SNV + SD**	0.79871	0.0414
**MSC + FD + SG**	0.82491	0.0386	**SNV + FD + SG**	0.81363	0.0395
**MSC + SD + SG**	0.98784	0.0193	**SNV + SD + SG**	0.98012	0.0214

**Table 4:** The influence of different spectral wavenumbers on the quantitative model.

Spectral Segment (cm^−1^)	R^2^	RMSECV	RMSEP	RPD
4000–5000	0.78352	0.0590	0.226	3.8356
5000–5400	0.85264	0.0409	0.187	4.5819
5600–6200	0.95347	0.0272	0.133	4.8934
6800–7400	0.84531	0.0420	0.195	4.6476
8200–9000	0.94310	0.0294	0.145	4.9456
5200–6700; 7700–8800	0.98784	0.0193	0.1064	5.5130

**Table 5:** The parameters of the model.

Item	Parameters
Method	PLS
Spectral Preprocess	MSC + SD + SG
Spectral Range	5200–6700 cm^−1^ and 7700–8800 cm^−1^
The Number of Factors	6
Determination Coefficient	0.9878
RMSEC	0.0193
RPD	5.5130
